# Construction Condition and Damage Monitoring of Post-Tensioned PSC Girders Using Embedded Sensors

**DOI:** 10.3390/s17081843

**Published:** 2017-08-10

**Authors:** Kyung-Joon Shin, Seong-Cheol Lee, Yun Yong Kim, Jae-Min Kim, Seunghee Park, Hwanwoo Lee

**Affiliations:** 1 Department of Civil Engineering, Chungnam National University, Daejeon 34134, Korea; kjshin@cnu.ac.kr (K.-J.S.); yunkim@cnu.ac.kr (Y.Y.K.); 2 Department of NPP Engineering, KEPCO International Nuclear Graduate School, Ulsan 45014, Korea; sclee@kings.ac.kr; 3 Department of Marine and Civil Engineering, Chonnam National University, Yeosu 59626, Korea; jm4kim@jnu.ac.kr; 4 School of Civil, Architectural Engineering and Landscape Architecture, Sungkyunkwan University, Suwon 16419, Korea; shparkpc@skku.edu; 5 Department of Civil Engineering, Pukyong National University, Busan 48513, Korea

**Keywords:** Keywords: load cell, optical fiber sensor, pre-stressed concrete, monitoring, pre-stress, damage

## Abstract

The potential for monitoring the construction of post-tensioned concrete beams and detecting damage to the beams under loading conditions was investigated through an experimental program. First, embedded sensors were investigated that could measure pre-stress from the fabrication process to a failure condition. Four types of sensors were installed on a steel frame, and the applicability and the accuracy of these sensors were tested while pre-stress was applied to a tendon in the steel frame. As a result, a tri-sensor loading plate and a Fiber Bragg Grating (FBG) sensor were selected as possible candidates. With those sensors, two pre-stressed concrete flexural beams were fabricated and tested. The pre-stress of the tendons was monitored during the construction and loading processes. Through the test, it was proven that the variation in thepre-stress had been successfully monitored throughout the construction process. The losses of pre-stress that occurred during a jacking and storage process, even those which occurred inside the concrete, were measured successfully. The results of the loading test showed that tendon stress and strain within the pure span significantly increased, while the stress in areas near the anchors was almost constant. These results prove that FBG sensors installed in a middle section can be used to monitor the strain within, and the damage to pre-stressed concrete beams.

## 1. Introduction

For the last half century, post-tensioned pre-stressed concrete (PSC) girders have been constructed due to the advantages and effectiveness of their structural behavior. To secure structural safety in post-tensioned PSC girders, it is very important to know the effective pre-stress force in the tendon [1,2,3]. Of the many studies and design specifications that have been proposed, there are several methods that can predict the pre-stress in a tendon of a PSC girder. However, because there are too many uncertainties related to the loss of pre-stress, there is always a huge gap between predictions and measurements.

Although it is important to know the effective pre-stress force in PSC girders, it is not easy to measure this during their years of service using conventional methods. Electrical strain gauges are difficult to install on tendons located inside the concrete and are easy to lose during the construction process [4,5]. Load cells embedded in the anchors of tendons are not available to measure the effective pre-stress force in the middle of PSC girders when the tendons are bonded. Therefore, information regarding effective pre-stress force is too limited. Generally, only the jacking force measured from the hydraulic unit during tensioning is available.

Various nondestructive test (NDT) methods have been studied to estimate the force of the pre-stressing tendons during the construction and service stages. However, most studies are limited to lab-scale applications. Specifically, there is practically no example in which an economically efficient estimation of the pre-stress force has been realized for the bonded pre-stressing tendons applied in existing pre-stressed concrete bridges [6].

The studies related to pre-stress measurement in bonded tendons can be classified based on the theory applied. The most famous approach is to use a guided wave or stress wave. However, this approach is limited to being applied to bonded pre-stressing steel filled with grout. The guided wave passing through the concrete cannot be measured practically due to its attenuation in concrete [7,8]. As an alternative to overcome the drawbacks of ultrasonic and stress waves, a technique using a magnetic field was studied. Wang et al. [9,10] developed magneto-elastic sensors that can monitor the stress in a multistrand cable for cable stayed or cable suspension bridges. However, most applications have been for cables and tendons not embedded inside the concrete [11,12].

Recently, optical fiber sensors have been developed and applied to monitor the pre-stress of tendons because of several advantages, such as high accuracy and electromagnetic interference resistance [13,14,15,16,17,18,19,20,21,22]. Previous studies reported that it is possible to measure the pre-stressing force of PSC structures by attaching an optical sensor to the surface of one of the strands used in such structures [16,17,18,19,20]. Distributed optical fiber sensing technology was also used to monitor the pre-stress of a tendon [21,22]. Fiber optic sensors can be attached directly to such strands, or in combination with a material similar to that of the strands.

As an application method of Fiber Bragg Grating (FBG) sensors, smart tendons with embedded FBG sensors have been developed to measure tendon strain directly [23,24]. By encapsulating the FBG sensor inside a seven-wire strand, the effective pre-stress force can be measured during construction and even during the service life of the product. In the literature, it was found that the effective pre-stress force can be accurately measured through FBG sensors, not only when tendons are directly exposed to the air [25,26], but also when tendons are embedded in concrete [27].

For this paper, the feasibility of monitoring the pre-stress of tendons during beam construction and of detecting damage to PSC girders that are in service was investigated experimentally. First, several kinds of sensors were tested and their appropriateness were verified. Next, PSC girders were fabricated with the sensors embedded. During their construction, the condition of the pre-stressing tendons was measured by the embedded sensors. Finally, girder stress was monitored while the applied load was increased until failure.

## 2. Validation Test of Sensors

### 2.1. Experimental Program

**Figure 1:**
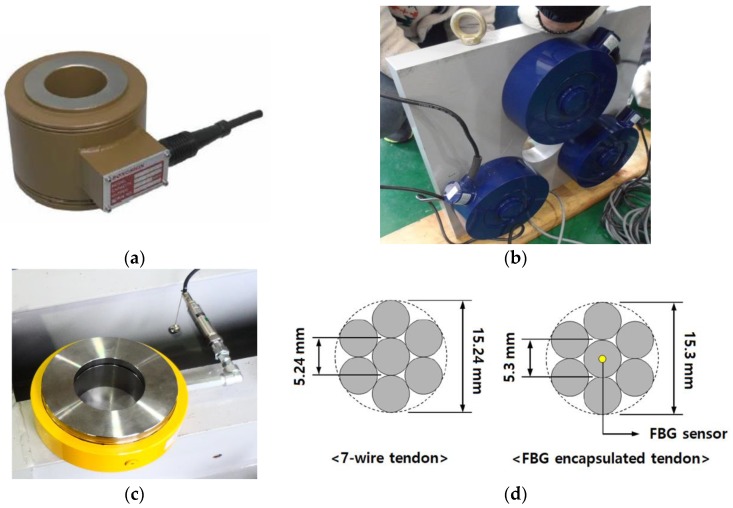
Sensors tested in this study. (**a**) Center-hole load cell; (**b**) Tri-sensor loading plate; (**c**) Hydraulic load cell; (**d**) Fiber Bragg Grating (FBG) sensor encapsulated seven-wire steel tendon.

As a first step, the sensors available with current technology were investigated, and it was verified that they could be used to measure the pre-stress of a tendon in a PSC girder. It should be noted that even though the alignment of the hydraulic jack, anchor head, and steel wire is adjusted carefully, the individual strands sustain difference prestress forces [12], which can lead to eccentric force on the anchor head. This eccentric effect cannot be avoided when a typical hydraulic jacking device is used. Four types of sensors were tested, as shown in Figure 1. Three of them were strain gauge-based force transducers, and the other was an FBG sensor.

#### 2.1.1. Center-Hole Load Cell

Center-hole load cells have been used widely to measure the pre-stress force directly. Because the sensor has a hole in its center, it is optimal for measuring the pre-stress of the tendon at the anchorage. However, it has been reported that sometimes the measured value is not as consistent or as precise as given in the specifications when an eccentric load is applied [25]. Therefore, this center-hole load cell needed verification as to whether it is appropriate for the measurement of pre-stress in a PSC girder. In this study, the effect of eccentricity on typical center-hole load cells was tested and investigated. The result indicates that such eccentricity leads to error in the measurements.

#### 2.1.2. Tri-Sensor Loading Plate

Because a center-hole load cell has high sensitivity to eccentricity, a new type of load measuring device was developed and tested. Three load cells were attached to a loading plate with a hole. The applied load on the plate could be measured by summing the loads of the three load cells. This loading plate was designed to minimize the effect of eccentricity based on mechanical theory. Even though there is eccentricity, the applied load can be measured precisely due to the statically determined structural characteristic of the loading plate. Figure 1b shows a schematic of the proposed loading plate with the load cells. The proposed tri-sensor loading plate was verified by experiment.

#### 2.1.3. Hydraulic Load Cell

Hydraulic load cells are force-balance devices that measure load as a change in the pressure of the internal filling fluid. The load, when applied to the surface area of the piston, causes a pressure increase in the hydraulic fluid. This pressure is transferred to the attached pressure transducer for measurement. Because the load is measured through hydrostatic pressure, it can be inferred that the measurements are unaffected by the eccentricity. Thus, a hydraulic load cell was tested as a candidate for the measurement of pre-stress with eccentricity.

#### 2.1.4. FBG Sensor

A Fiber Bragg Grating (FBG) sensor is a type of distributed Bragg reflector constructed in a short segment of optical fiber that reflects particular wavelengths of light and transmits all others. This is achieved by creating a periodic variation in the refractive index of the fiber core, which generates a wavelength-specific dielectric mirror. An FBG can therefore be used as an inline optical filter to block certain wavelengths, or as a wavelength-specific reflector. Based on these principles, an FBG sensor can be used to measure a strain profile of a tendon. In this study, an FBG sensor was tested for possible application to pre-stress measurement. In order to measure the strain of a tendon, a special tendon with an embedded FBG sensor was made in the lab [23].

### 2.2. Test Method and Equipment

The sensors listed in Section 2.1 measure load using different principles and mechanisms. To compare the various types of sensors for the purpose of measuring pre-stress, the sensors should be calibrated on the same basis. Therefore, all the sensors used in the experiment were calibrated and compared in the same test machine. The piece of equipment used for the calibration was a fatigue material testing machine manufactured by MTS. The sensors were tested and calibrated with the load cell built into the MTS testing machine. The load cells were calibrated based on compressive load while tensile force was applied on the FBG sensors.

**Figure 2:**
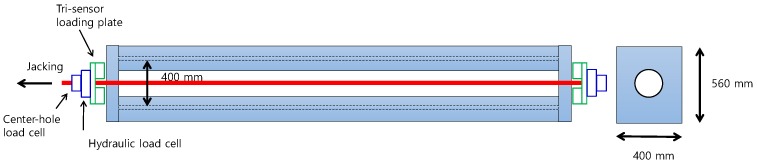
Steel frame of a girder for validation test (not to scale, stiffeners not shown).

**Figure 3:**
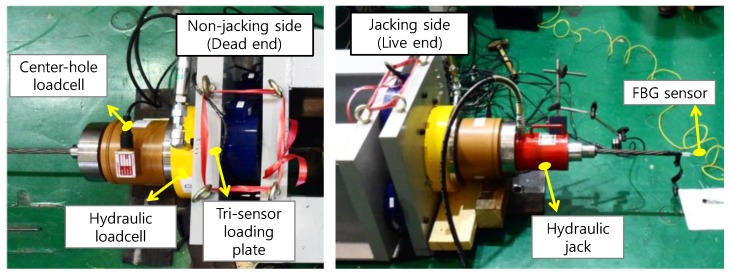
Installation of sensors at the ends of the steel frame.

In order to measure all of the sensors at once with the same loading condition, a small-scale girder was prepared. Instead of using a PSC girder that has friction between tendons and sheath, a steel girder was made with two I-shaped steel girders connected together, as shown in Figure 2. The girder had a large interior space so that no friction would occur when the tendons were installed inside the girder. As shown in Figure 2, the size of the model was 560 × 400 × 5000 mm and it had two anchor plates, each with a circular hole in which to place the two ends of the pre-stressing tendon. At the ends of the girder, the sensors needed for verification were installed, as shown in Figure 3. All of the sensors were calibrated in advance to improve the credibility of the results.

After the test setup was completed, one pre-stressing tendon was installed. Then, the pre-stress was applied to the tendon with a hydraulic jack until the stress reached about 70% of maximum strength. The maximum load applied to a single tendon was about 160 kN.

The load was increased from zero to a target value in stages. During this loading process, the sensors installed on the girder were monitored. The sensors based on the strain gauge principle were monitored continuously during the entire loading process using a National Instruments (NI) data acquisition system. Also, the strain was calculated from the wavelength change of the FBG sensor measured using an interrogator. The temperature of the specimens was recorded and used for the consideration of the temperature effect of FBG sensors. The coefficients and constants for the FBG sensors were the same as those of Kim et al. [24].

### 2.3. Test Results

#### 2.3.1. General Behavior

During the pre-stressing process, the load applied to the specimen was monitored using a center-hole load cell, a tri-sensor loading plate, and a hydraulic load cell. Among these three load cells, the measurement from the tri-sensor loading plate was selected as the reference load. This is because the mechanical behavior of this load cell is determinate and stable so that the eccentricity of the load cannot influence its measurement of the total pre-stress. During the entire test, the reference load showed values consistent with the pressure of the hydraulic jack.

**Figure 4:**
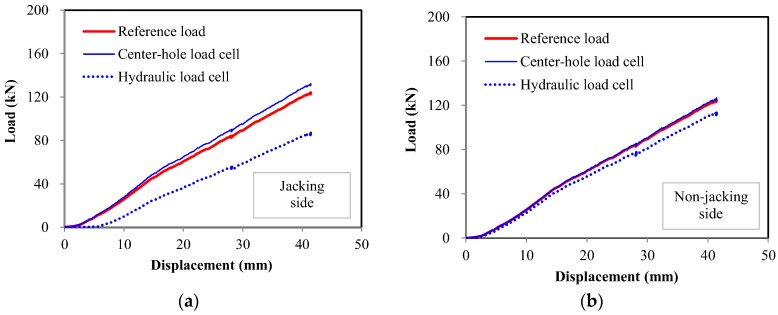
Test results of one tendon in the steel frame. (**a**) Jacking side; (**b**) Non-jacking side.

Figure 4 shows a result for one tendon. In the figure, the displacement means the movement of the hydraulic pre-stressing jack. Figure 4a shows the results measured at the jacking side (live end), while Figure 4b shows the results at the opposite side (dead end). The results show that the measurements from the dead end had relatively lower variation than those from the live end. The figures show that the hydraulic load cell underestimated the pre-stress, while the center-hole load cell overestimated it.

The R-square values were above 0.997 when linear regressions were conducted for each measurement. However, the slope of the fitted line varied with the load cell used. This means that the data from all of the tested load cells can be considered linear, but the accuracy varies with the type of load cell.

#### 2.3.2. Center-Hole Load Cell

**Figure 5:**
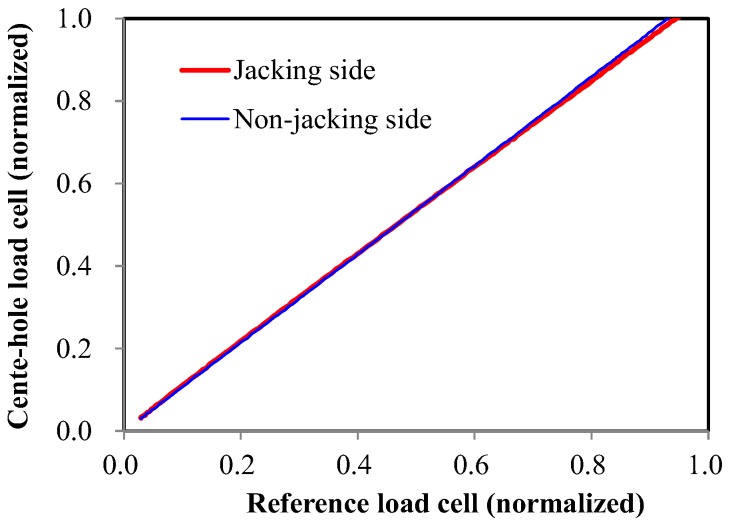
Relationship of the measurements between reference and center-hole load cells.

When the center-hole load cells were calibrated for a centric load, the calibration results were sufficient to provide the linearity and repeatability specified by the manufacturer. However, the results shown in Figure 4 show different patterns. The linearity of the results was sufficient, but the measurements were overestimated, as shown in Figure 5. This trend coincides with previous reports [25] that the measured value is not consistent with that given in the specifications when an eccentric load is applied to a center-hole load cell.

**Figure 6:**
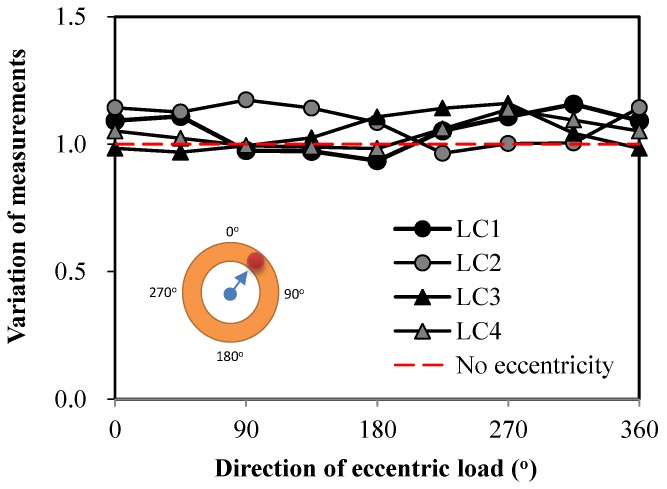
Variations of measurements in relation to the direction of eccentric loads.

Therefore, in order to investigate this characteristic intensively, the influence of eccentricity on the measurement was tested. First, the load was applied to the center-hole load cell without eccentricity. Then, the load was applied to the same load cell with various eccentric configurations. The directions of loading point varied from the center of the load cell, while the distance from the central point was maintained as shown in Figure 6.

Four load cells were tested and the test results are shown in Figure 6. The variations are shown as the ratios between the measurement from the load cell and the applied load on the load cell. It needs to be noted that the loads applied by the MTS test machine were almost the same values.

For a centric load, the ratio is equal to ‘1’. However, Figure 6 indicates that the eccentric measurement varied as the location of the load changed, even though the same load was applied. Most of the eccentric measurements were larger than the applied load, while some were smaller. This can be interpreted to mean that the measurements from the center-hole load cell were overestimated or underestimated when an eccentric load was applied. This trend can be considered not limited to this product, but a typical characteristic of a center-hole load cell. In the center-hole load cell, the load is supposed to be transferred through the whole body. However, the load is typically evaluated using only a few strain gauges attached to the body, so errors can be induced when the strain on the body is not uniform. Therefore, special attention needs to be paid when a prestress is measured using a center-hole-type load cell.

In brief, the center-hole load cell is the most commonly used transducer for the measurement of pre-stress. However, it generates errors when an eccentric load is applied. The magnitude of eccentricity and error did not show any particular tendency, but in most cases, the value indicated by the center-hole load cell was larger than the reference load. Therefore, careful attention should be paid when using a center-hole load cell.

#### 2.3.3. Hydraulic Load Cell

As done for the center-hole load cell, the hydraulic load cells were calibrated first for a centric load, and then tested for validation. The calibration result showed linearity and repeatability for a centric loading condition. However, Figure 4 shows that the measured value from the hydraulic load cell was different from the expected value.

**Figure 7:**
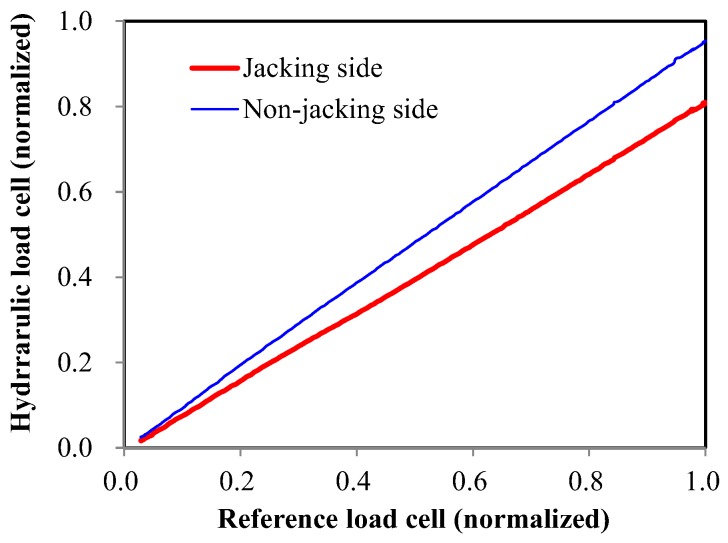
Relationship of the measurements between reference and hydraulic load cells.

Figure 7 shows the characteristics of the hydraulic load cell. It can be observed that the loads measured from either the jacking or non-jacking side were less than the reference load. In addition, these differences were much larger on the jacking side. In pre-stressed concrete structures, the eccentricity of the pre-stress cannot be avoided due to the misalignment of the hydraulic jack, anchor head and steel wire, and due to variation of each tendon‘s stress [12]. In addition, this eccentricity is much larger on the jacking side than on the non-jacking side. Thus, it can be concluded that the eccentricity of the applied load influenced the characteristics of the hydraulic load cell due to the inherent structural characteristics of the sensor.

A pressure-loaded load cell has clearance for its piston to move, and there are interior oil rings to prevent the leakage of oil. Therefore, when an eccentric load is applied, it could be expected that the hydraulic piston would rotate a little within its clearance, thereby causing loss due to the frictional force between the piston and the outer wall. In particular, the pressure-type load cell manufactured in this study was considered to be more vulnerable to eccentricity, because the axial length of the piston was short (low height) so that the probability that rotational deformation would occur was high. Thus, it can be concluded that pressure-type load cells without sufficient height to prevent rotation of the cylinder are not suitable for the measurement of pre-stressing force.

#### 2.3.4. Tri-Sensor Loading Plate

The tri-sensor loading plates were calibrated and tested. Three individual compressive load cells attached to the tri-sensor loading plate were calibrated first. Then, the tri-sensor loading plates were assembled and tested for validation. The results show that the measures from each load cell varied in relation to the eccentricity. As the eccentricity increased, the standard deviations of the measurements from each load cell increased. However, the summation of the measurements did not change regardless of the eccentricity. Thus, it was deduced that the tri-sensor loading plate could provide a reference load not influenced by eccentricity.

#### 2.3.5. FBG Sensor

**Figure 8:**
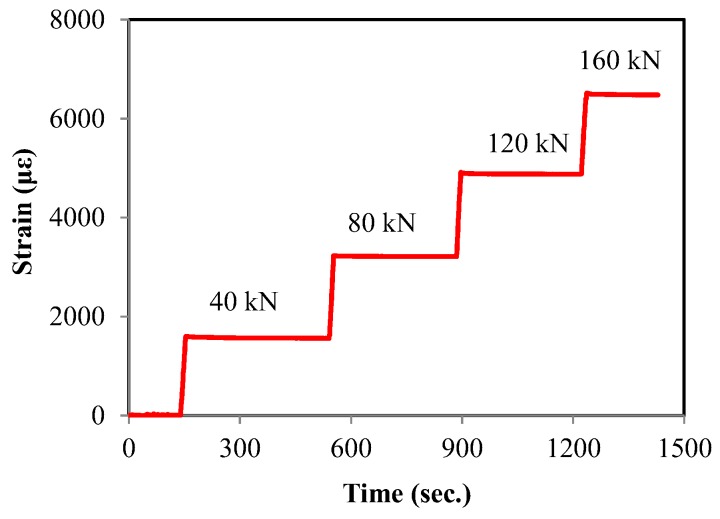
Strain measured by FBG sensor.

Figure 8 shows the results of the FBG sensor measurement. In this case, the strain is calculated from changes in the wavelength from a sensor. The result shows that the strain increased in relation to the increase of the tensile force of the pre-stressing tendons. The results prove that the strain increased proportionally as the applied pre-stress increased. Thus, it can be concluded that the applied load can be estimated from the measured strain using the relationship between the strains measured and the forces applied. The greatest advantage of this FBG sensor is that the strain at any location inside the tendon can be measured, while the other load cells can only measure the stress at the end of a PSC beam.

## 3. Construction Process and Damage Monitoring of PSC Girders

### 3.1. Experimental Program with PSC Girders

**Figure 9:**
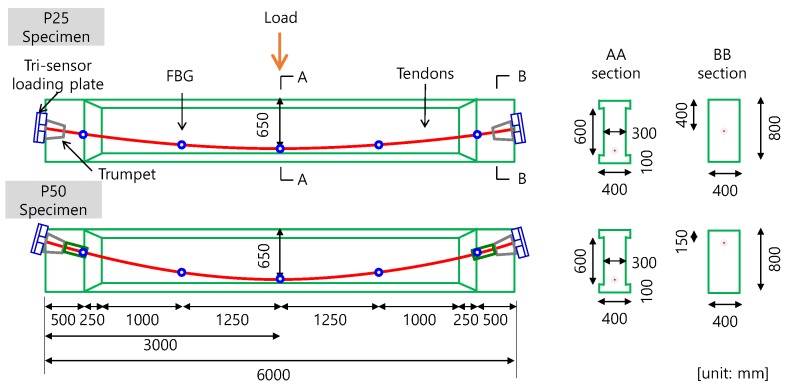
Details about the post-tensioned girder specimens and sensors installed. P25, eccentricity of tendon profile set to be 250 mm; P50, eccentricity of tendon profile set to be 500 mm.

In order to evaluate the feasibility of monitoring the pre-stress during the construction stage and monitoring the damage to the PSC girder during a service stage, a structural beam member intended to represent the behavior of a typical PSC girder was designed and fabricated [28]. For monitoring purposes, load cells and FBG sensors were installed as shown in Figure 9.

Tri-sensor loading plates were installed at both (live and dead) ends of the beam in order to measure the pre-stressing forces. The live anchor refers to the end where the pre-stressing is applied, and the dead anchor means the opposite end where pre-stress is not applied. Because the pre-stress inside the beam cannot be measured using typical load cells, a smart tendon with an FBG sensor was installed to monitor the strain distribution of the tendon.

Two post-tensioned girders were fabricated. The dimensions of the girders (height and length) were 0.8 and 6 m, respectively. The web thickness was 300 mm. The test variable was the tendon profile: the eccentricity of the tendon profile was set to be 250 mm (P25) or 500 mm (P50). Each specimen contained three conventional 15.2 mm seven-wire strands and one smart tendon, in which the FBG sensor was embedded. Reinforcing bars of 1.28% longitudinal reinforcement ratio and 0.23% shear reinforcement ratio were embedded. The compressive strength determined in a loading test was 45 MPa. To measure tendon strains during the test, five measuring points were set along the FBG sensors in each specimen. Figure 9 shows details about the dimensions of the specimens, tendon profiles, and locations of the FBG sensors.

The specimens were made using the same procedures as those for constructing the PSC girders at the site. First, the assembly of reinforcing bars and sheaths for the entire specimens was completed. Concrete was cast and cured. Then, the seven-wire strands were tensioned using a hydraulic jack and a pump. The pre-stress was applied to the live anchor. During these construction procedures, the pre-stress was monitored in real time using the installed sensors.

After the specimens were stored for four months, the specimens were loaded using a 3-point loading method with a hydraulic actuator of which the capacity was 3000 kN, as presented inFigure 9. The pure span was set to be 4.5 m to observe any difference in tendon strain between the damaged zone within the pure span and the undamaged zone beyond the pure span. During the test, the tendon strain was measured throughout using the embedded FBG sensors, and the pre-stress force was measured through the load cells.

### 3.2. Test Results: Construction Stage

#### 3.2.1. Pre-Stressing Stage

**Figure 10:**
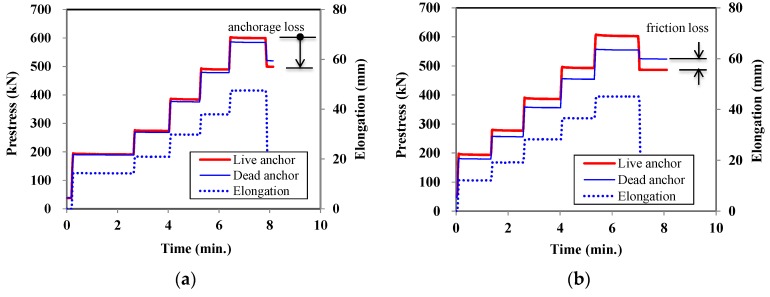
Pre-stress and elongation measured at the anchorage. (**a**) P25 specimen; (**b**) P50 specimen.

During the construction process, pre-stress was applied to the tendon and transferred to the concrete beam. The pre-stress at the live anchor, pre-stress at the dead anchor, and elongation of the tendon were measured during the jacking process, as shown in Figure 10. As seen in Figure 10, the pre-stress at the dead end and elongation increased with an increase in the pre-stress applied to the live end. There were regions where the measured values were constant, because the pre-stressing was applied in several steps.

The pre-stress measured at the live end coincided well with the applied pressure measured at the hydraulic jack. The measured load at the dead end was smaller than that at the live end due to friction losses that occurred in the tendon. The gap between loads at the live and dead anchors varies with respect to the test specimens. As the curvature of the tendon profile increased, the difference increased too. From Figure 10, it was calculated that the measured friction loss through the tendon was 4.2% and 7.7% for the P25 and P50 specimens, respectively.

After applying pre-stress to the tendon, the pre-stress was released and transferred to the anchorage and concrete. During this process, a pre-stress loss occurred due to the anchorage-seating. From the measurements, it was observed that the anchorage-seating loss of live anchor was 17% and 19% for the P25 and P50 specimens, respectively.

From the load cell measurements, it was shown that the pre-stressing force at both ends of the beam can be measured so that the construction process and quality of a PSC girder can be monitored successfully. The pre-stress losses, which occur during the construction process, can be evaluated from exterior measurements. However, the stress or strain inside the beam cannot be measured directly using the load cells.

**Figure 11:**
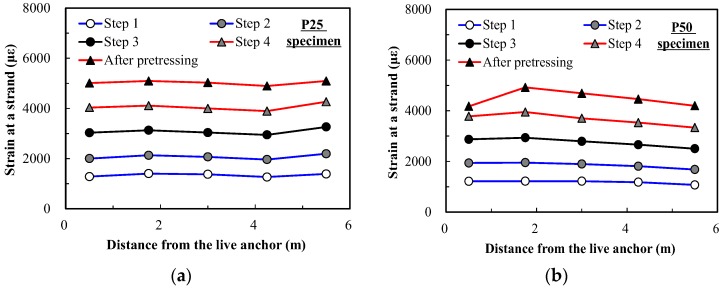
Strain measured by embedded FBG sensors. (**a**) P25 specimen; (**b**) P50 specimen.

Figure 11 shows the measurements from the FBG sensors during the process of jacking the tendon. Figure 11a shows that the strains along the tendon increase as the applied load increases, as expected. However, Figure 11b shows that the strain near the live anchor decreased in the last stage due to unexpected slippage of the tendon. This kind of small change cannot be monitored using only a load cell. This figure proves that the strain profile of a tendon located inside a concrete beam can be monitored successfully using FBG sensors. Even local changes of strain can be monitored.

Figure 10 and Figure 11 prove that the load cells are able to measure the pre-stress of a tendon at the anchorage, and that FBG sensors can measure the strain at any location of the tendon where FBG sensors are embedded. The pre-stress of tendons during prestressing stage can be monitored successfully with these load cells and FBG sensors.

#### 3.2.2. Yard Storage Stage and Pre-Stress Losses

After the beams were fabricated and pre-stressed, they were stored for four months. After this, the beams were tested for structural performance and damage monitoring. After pre-stressing was applied to the tendon, the pre-stress on the tendon varied with time due to several causes, such as elastic shortening, creep, and shrinkage of the concrete. Calculation of the effective pre-stress that remains in a tendon plays a major part in the design stage of PSC beams, because the amount of applied pre-stress is a key factor governing the structural performance of a PSC beam. The pre-stress losses can be evaluated using well-known theories or design codes [1,2,3]. However, it is not easy to know the actual pre-stress applied to the tendon in the construction or in-service stages.

Table 1 shows the summarized pre-stresses that were monitored using the embedded load cells and FBG sensors. In the table, LL and LD represent the load cell at the live anchor and dead anchor, respectively. This table shows that the actual pre-stress on the tendon can be monitored rationally with embedded sensing technology. The calculated losses can be used to prove (or improve) the design process and construction quality of PSC beams.

### 3.3. Test Results: Loading Stage

#### 3.3.1. General Behavior

**Figure 12:**
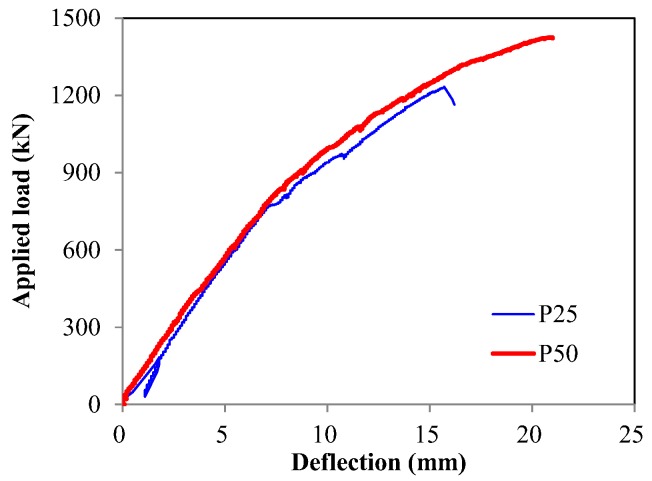
Load-center deflection response.

As the applied load increased, the deflection increased, as shown in Figure 12. The crack patterns were recorded and checked at various loading steps until the specimens failed. During the test, flexural cracks occurred at the bottom of the center, and then flexural-shear cracks were induced as the applied load increased. Subsequently, web-shear cracks were formed. As a result, all of the specimens showed shear failures with a dominant diagonal crack. Detailed crack patterns of the specimens can be found in the study of Lee et al. [28].

#### 3.3.2. Test Results: Pre-Stress Forces and Damage

During the loading process, the pre-stress of the tendon was measured through the load cells. However, the pre-stress forces measured at the ends of the beam changed very little; the changes measured were less than 1% of the initial values.

Generally, the tendons in a PSC girder are grouted for several reasons, such as protection against corrosion and improvement of structural integrity. After tendons are grouted and bonded, the tendon and concrete beam behave as a single structure, so that there is no increment of strain at the ends of a beam where external force has no influence.

Thus, it can be concluded that load cells, which can measure the pre-stress only at the ends of a beam, are not appropriate for evaluation of damage to PSC girders, because no noticeable differences were measured using such sensors.

#### 3.3.3. Test Results: Tendon Strain and Damage

**Figure 13:**
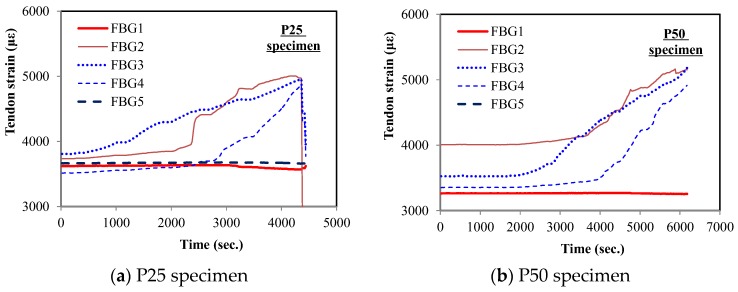
Tendon strain of specimens in relation to time.

The tendon strain measured through the FBG sensors during the test is presented over time in Figure 13. At the beginning of the loading test, the tendon strain was around 0.003–0.004, which decreased by about 0.0007 from the value measured just after post-tensioning. This was due to the effect of concrete creep and shrinkage. As the load was applied at the center of the girders, the tendon strains at FBG1 and FBG5 (located near the anchors) showed very little variation, even under failure. This indicates that damage caused by flexural cracks in PSC girders cannot be detected with sensors embedded at the anchors.

On the other hand, tendon strains at the sensing points within the pure span exhibited significant increase as the applied load increased and cracks occurred. Because flexural cracks were formed at the bottom of the center, the tendon strain at FBG3 (near the first flexural crack) started increasing first while the others remained almost constant. Then, the tendon strains at FBG2 and FBG4 increased later as the number of cracks increased and the damage increased. This indicates that damage due to flexural cracks in PSC girders can be detected by the strain increment monitored by FBG sensors near cracks. In addition, it can be inferred that FBG sensors installed in appropriate regions would make it possible to evaluate how much a PSC girder is damaged.

## 4. Conclusions

Pre-stressing force has not been managed after construction, even though it is a very important factor for maintaining the structural safety of PSC girder bridges. Usually, the pre-stressing force is measured only during construction using a jacking device, and after that it cannot be managed practically. For this reason, in this study, the measurement of pre-stress was investigated using embedded sensors that are currently available, with the goal of proposing ‘smart’ pre-stressed concrete girders that could monitor the pre-stress of the tendon and damage of a beam from its birth to the end of its life.

Four types of sensors were installed on a small steel frame, and the applicability and accuracy of those sensors were tested while pre-stress was applied to the frame. The results show that the hydraulic load cells have a tendency to underestimate the pre-stress when it is loaded with eccentricity. The center-hole load cell shows irregular error in the presence of the eccentricity. The tri-sensor loading plate and FBG sensors measure the pre-stress successfully.

In order to investigate the feasibility of monitoring the pre-stress of tendons in construction and service stages, 6 m-long post-tensioned PSC girders were fabricated. During the construction process, the pre-stress applied to the tendons was monitored. The results prove that the pre-stressing history and construction quality can be monitored precisely using embedded sensors. Pre-stress losses can be evaluated using the monitored results.

Through a 3-point loading test for the PSC girders, tendon stress and strain were measured by embedded sensors. The measurements prove that the stress and strain of tendons near the anchors change only a little, even after failure. However, it was observed that the FBG sensors at the middle section showed significant increases due to flexural deformation and cracks within the pure span. Consequently, it can be concluded that damage due to flexural deformation and cracks in PSC girders can be detected by FBG sensors at the location where the damage occurs.

## Figures and Tables

**Table 1:** Results from monitoring pre-stress and corresponding pre-stress loss.

**P25 Specimen**	**LL (kN)**	**FBG1 (με)**	**FBG2 (με)**	**FBG3 (με)**	**FBG4 (με)**	**FBG5 (με)**	**LD (kN)**
Initial (A)	0	0	0	0	0	0	0
Jacking (B)	600	5009	5091	5030	4894	5092	585
After anchoring (C)	500	4153	4292	4342	4255	4555	520
Before loading test (D)	467	3682	3532	3824	3753	3645	479
Jacking loss (1-B/C)	16.7	17.1	15.7	13.7	13.0	10.6	11.1
Long term loss (1-D/C)	6.6	11.3	17.7	11.9	11.8	20.0	7.9
**P50 Specimen**	**LL (kN)**	**FBG1 (με)**	**FBG2 (με)**	**FBG3 (με)**	**FBG4 (με)**	**FBG5 (με)**	**LD (kN)**
Initial (A)	0	0	0	0	0	0	0
Jacking (B)	603	4172	4919	4687	4459	4195	555
After anchoring (C)	487	3038	3900	3933	4153	4000	524
Before loading test (D)	468	2790	3348	3517	4000	3253	464
Jacking loss (1-B/C)	19.2	27.2	20.7	16.1	6.9	4.7	5.6
Long term loss (1-D/C)	3.9	8.2	14.2	10.6	3.7	18.7	11.5

LL: load cell at live anchor; LD: load cell at dead anchor.
